# Accuracy of Surgical Guides in Guided Apical Surgery: An In Vitro Comparative Study

**DOI:** 10.3390/dj13120561

**Published:** 2025-12-01

**Authors:** Nancy Soraya Romero Mora, Maria Soledad Peñaherrera Manosalvas, Henry Paul Valverde Haro

**Affiliations:** 1 Postgraduate Program in Dentistry, Universidad de los Hemisferios, Quito 170527, Ecuador; nsromerom@estudiantes.uhemisferios.edu.ec (N.S.R.M.); mspenaherreram@uhemisferios.edu.ec (M.S.P.M.); 2 Program in Dentistry, Universidad Nacional de Chimborazo, Riobamba 060110, Ecuador

**Keywords:** cone-beam computed tomography, 3D printing, apicoectomy, computer-assisted surgery, endodontics, surgical guides

## Abstract

**Background/Objectives:** Guided endodontic microsurgery is a novel approach designed to improve safety and precision compared with conventional freehand techniques. The present study aimed to evaluate the accuracy, stability, and operative time of trephinations performed using stereolithographic surgical guides designed with Blue Sky Plan and Exoplan software compared with the conventional technique. **Methods:** A comparative in vitro study was conducted on 72 roots from 12 stereolithographic mandibles. Cone-beam computed tomography (CBCT) and intraoral scans were superimposed to design 16 surgical guides with verification windows and metallic sleeves. Trephinations were performed using a control freehand group, Blue Sky Plan, and Exoplan. Stability, accuracy, and operative time were assessed. Statistical analysis included ANOVA, Kruskal–Wallis, and chi-square tests. **Results:** Exoplan demonstrated superior accuracy (0.17 mm deviation), stability (12.5% failures vs. 50% in Blue Sky Plan), and shortest operative time (106 s vs. 127 s and 155 s). Differences were statistically significant (*p* < 0.05). Operative accuracy was independent of surgical duration. **Conclusions:** Exoplan outperformed Blue Sky Plan and freehand trephinations in stability, accuracy, and efficiency. These findings highlight the potential of digital guides for safer and more predictable endodontic microsurgery. Further clinical validation is required.

## 1. Introduction

Endodontic microsurgery is often required when conventional treatment or retreatment fails. Despite technological advances, apical surgery remains complex due to restricted surgical fields and the proximity of critical anatomical structures [1,2].

Recent innovations, such as cone-beam computed tomography (CBCT) and magnification systems, have improved diagnostic and surgical precision [3]. However, freehand procedures remain challenging, especially for less experienced clinicians, as they require real-time interpretation of radiological data during surgery [4,5].

The introduction of guided endodontics (GE) in 2016 allowed integration of CBCT and intraoral scans with 3D printing to design surgical guides [6,7]. These guides demonstrated improvements in precision, reproducibility, and safety in a range of scenarios [8]. Case reports and systematic reviews have confirmed their value in challenging cases such as pulp canal obliteration or traumatic injuries [9,10].

Static guided endodontics (SGE) has been validated in both in vitro and clinical contexts, while dynamic navigation systems have been developed to provide additional flexibility [11,12]. Both modalities aim to reduce operator variability, although static systems offer more reproducible and cost-effective workflows [13,14].

The present study aimed to compare stability, accuracy, and operative time in apical trephinations performed with Blue Sky Plan, Exoplan, and conventional freehand methods. We hypothesised that software-assisted guides would significantly outperform freehand procedures, and that Exoplan would demonstrate superior accuracy and efficiency compared with Blue Sky Plan [15,16,17].

## 2. Materials and Methods

### 2.1. Study Design and Ethical Approval

This was an experimental, comparative in vitro study using stereolithographic mandibular models. Ethical approval was granted by the Ethics Committee of the Postgraduate Programme in Dentistry, Universidad de los Hemisferios (CEUHE25-40; 5 May 2025). Anonymised CBCT datasets were obtained from the institutional teaching repository, with donors having provided prior written consent for research use.

### 2.2. Sample and Teeth Selection

Twelve mandibular models with complete dentitions were fabricated, yielding 72 roots (six per model). The planned sites included central incisors, canines, premolars, and mesial and distal roots of first molars. A ≥2 mm safety margin was respected for the inferior alveolar nerve canal and mental foramen in all cases to ensure anatomical safety.

### 2.3. Surgical Guide Design and Software Versions

**Figure 1:**
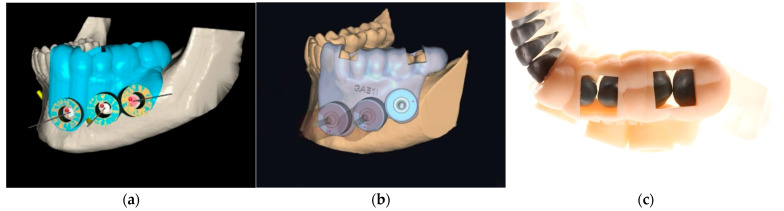
Surgical Guide Design: (**a**) Conventional apical trephination without surgical guides; (**b**) Trephination with guides designed in Blue Sky Plan; (**c**) Verification windows (9.5 × 12 mm) on proximal ridges of premolars and molars.

Surgical guides were designed using Blue Sky Plan v4.12.13 (Blue Sky Bio, LLC, Libertyville, IL, USA) and Exoplan v3.1 Rijeka (Exocad GmbH, Darmstadt, Germany) (Figure 1a). Both programs integrated CBCT (Hyperion X9, Myray, Imola, Italy) with STL surface scans (PrimeScan, Dentsply Sirona, Bensheim, Germany). Guides incorporated occlusal and buccal support surfaces (Figure 1b), as well as verification windows measuring 9.5 × 12 mm (Figure 1c). Stainless steel sleeves were embedded (inner diameter 4.25 mm, height 5 mm) with an offset to achieve a 3 mm apical resection.

### 2.4. Guide Fabrication and Quality Control

Sixteen guides were printed using Water-Wash Resin+ (Anycubic Technology Co., Ltd., Shenzhen, China), with identical printing protocols applied across groups to avoid bias. Post-processing included a two-stage isopropyl alcohol wash and light curing at 60 °C for 20 min (Wash and Cure 2.0, Anycubic Technology Co., Ltd., Shenzhen, China). Dimensional accuracy was verified using calibrated pin gauges (tolerance ± 0.02 mm) and inspection of occlusal supports (tolerance ± 0.10 mm).

### 2.5. Trephination Procedure

**Figure 2:**
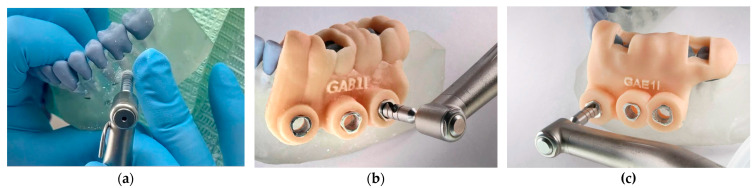
Trepanation using the three evaluated methods: (**a**) Conventional apical trephination without surgical guides; (**b**) Trephination with guides designed in Blue Sky Plan; (**c**) Trephination with guides designed in Exoplan.

Trephinations were performed with a surgical motor (NSK Surgic Pro, Nakanishi Inc., Kanuma, Japan) using sterile 3.5 mm internal diameter trephine burs. Each bur was reused for a maximum of three perforations, completing 24 apicoectomies per group. This standardised protocol minimised variability due to bur wear. The planned osteotomy corresponded to a 3 mm apical resection; the trephine offset of 0.5 mm preserved adjacent bone structure. In guided groups, metallic sleeves constrained angulation and depth with drill stops, ensuring precise reproduction of the virtual trajectory (Figure 2).

### 2.6. Operator Calibration and Randomisation

Three operators underwent joint calibration through pilot training. Randomisation of experimental groups followed a Latin-square design to avoid operator bias. Duplicate measurements were taken by two blinded observers one week apart, achieving intraclass correlation coefficients above 0.90.

### 2.7. Statistical Analysis

Statistical analysis was performed using Microsoft Excel (Microsoft Corporation, Redmond, WA, USA) and SPSS v26 (IBM Corp., Armonk, NY, USA). Normality was assessed using the Shapiro–Wilk test, followed by ANOVA or Kruskal–Wallis for continuous variables, and chi-square tests for categorical outcomes. The significance threshold was set at α = 0.05.

## 3. Results

### 3.1. Stability

In the control group (G1), no failures were observed. In the Blue Sky Plan group (G2), four out of eight guides exhibited mobility greater than 0.5 mm, corresponding to a failure rate of 50.0%. By contrast, only one guide in the Exoplan group (G3) showed instability (12.5%). Although chi-square analysis did not reveal statistically significant differences between groups (*p* > 0.05), the confidence intervals suggest a clear trend toward improved stability with Exoplan (Table 1).

### 3.2. Accuracy

Deviation from the planned 3 mm apical resection was highest in the control group (mean = 1.16 ± 0.82 mm), followed by Blue Sky Plan (0.83 ± 0.58 mm). Exoplan demonstrated the greatest precision, with a mean deviation of only 0.17 ± 0.20 mm. The Kruskal–Wallis test confirmed significant differences between groups (*p* = 0.000). Overall, Exoplan not only reduced variability but also maintained resection lengths closer to the digital planning (Table 2).

### 3.3. Operative Time

Trephination times were longest in the control group (mean = 154.6 ± 38.6 s), intermediate with Blue Sky Plan (127.5 ± 34.0 s), and shortest with Exoplan (106.5 ± 22.8 s). Statistical analysis (Kruskal–Wallis, *p* = 0.000) confirmed significant differences among all groups, indicating that higher precision did not translate into longer operative times; rather, the most accurate guides also yielded the fastest procedures (Table 3).

### 3.4. Correlation Analysis

No significant correlation was found between the degree of deviation and operative time (*p* > 0.05), suggesting that improvements in accuracy are independent of procedural duration.

## 4. Discussion

This study compared the stability, accuracy and operating time of trepanations performed using the Exoplan, Blue Sky Plan and conventional freehand methods. Exoplan achieved the best overall performance, thus confirming our hypothesis that software-assisted guides can improve the outcomes of guided apical surgery.

The superior accuracy of Exoplan can be attributed to its refined data integration and design features, which stabilise guide placement and minimise registration error. These results align with previous reports on the high accuracy of static guided endodontics, with deviations ranging from 0.1 to 0.3 mm [6,18,19]. In contrast, freehand resections typically exceed 1 mm, corroborating the results published by Peng et al. (2021) [20] and Huth et al. (2024) [13].

The shorter operating time observed with Exoplan was not only due to digital planning, but also related to the geometry of the guide. The locking tube’s design prevented micro-movements and enabled uninterrupted drilling; in contrast, the cylindrical design of the Blue Sky Plan required repeated adjustments. This is consistent with the observations of Zhao et al. (2023) [15], who demonstrated that innovations in guide sleeve design can significantly reduce procedure duration. Similarly, Cabezón et al. (2023) [17] confirmed that optimised static guide designs can achieve comparable efficiency to dynamic navigation systems.

Errors in surgical precision should not be attributed solely to software performance. Pilot tests in this study revealed that resin fragility and poor fitting of metal sleeves could compromise results. This is consistent with the findings of Strbac et al. (2017) [21] and Moreno-Rabie et al. (2020) [9], who emphasised the impact of material properties and manufacturing quality on clinical viability. Therefore, high-quality 3D printing protocols and rigorous quality control are essential to ensure the success of guided microsurgery.

From a clinical perspective, these findings suggest that Exoplan has the potential to enhance the safety and predictability of endodontic microsurgery, particularly for less experienced clinicians. This is consistent with systematic reviews [12,22,23] and recent cadaver studies [18] advocating the use of guided systems to reduce operator variability and improve outcomes. Importantly, our results showed no correlation between accuracy and operative time, indicating that improved accuracy does not necessarily prolong procedures, as was previously assumed [24].

However, some limitations must be acknowledged. Firstly, the present study was conducted in vitro, excluding biological factors such as soft tissue interference, bleeding and patient movement, which may affect guide stability. Additionally, the relatively small sample size limited statistical power, particularly for categorical outcomes. Therefore, future research should include larger clinical trials and direct comparisons between static and dynamic navigation systems [11,12]. Hybrid workflows that combine the precision of static navigation with the flexibility of dynamic navigation may represent the next step in guided microsurgery [22,25].

Recent advances in guided endodontic microsurgery demonstrate a consistent trend towards greater precision, safety and minimally invasive access compared to conventional techniques [26,27]. Authors such as Antal et al. (2019, 2020) [28,29] and Sutter et al. (2019) [30] have introduced digitally planned static guides and customised trephine burs to achieve precise apical resections with reduced operating time and better control of osteotomy dimensions.

Several studies have validated the accuracy of 3D-printed surgical guides in both cadaveric and in vitro models, highlighting their reliability in clinical applications [31,32]. Subsequent developments, such as dynamic navigation and augmented reality systems, have improved the precision and usability of guided apicoectomies [33]. Comparative analyses suggest that trephine-guided microsurgery allows for smaller osteotomies and more conservative bevel angles while maintaining procedural efficiency [34,35].

These findings are corroborated by clinical reports, which emphasise the advantages of improved surgical visualisation and greater predictability when accessing the apex [7,36,37]. Similarly, recent reviews and consensus reports emphasise that guided endodontic microsurgery represents a significant development in endodontic practice [38,39,40]. This approach integrates CAD/CAM digital planning, customised workflows and patient-specific instruments [2,23,34]. These improvements enhance accuracy and clinical outcomes. Taken together, these studies demonstrate that digitally guided apicoectomy is evolving from an experimental innovation into a reproducible, evidence-based clinical standard.

## 5. Conclusions

Exoplan surgical guides demonstrated superior stability, accuracy, and efficiency compared with Blue Sky Plan and conventional freehand techniques in guided apical surgery. These findings support the clinical adoption of digitally designed guides to enhance safety and predictability. Further clinical trials with larger samples are required to validate these results under real operative conditions.

## Figures and Tables

**Table 1:** Stability results of surgical guides.

Group	n (Models)	Failures (+)	Proportion (%)	95% CI for Failures
G1 Control	8	0	0.0	–
G2 Blue Sky	8	4	50.0	21.5–78.5
G3 Exoplan	8	1	12.5	0.7–53.3

Chi-square test: *p* > 0.05 (no significant association).

**Table 2:** Descriptive statistics of deviation from planned 3 mm apical resection (mm).

Group	Samples (n)	Minimum	Maximum	Median	Mean	SD	CV (%)
G1 Control	24	−0.77	3.14	1.16	1.16	0.82	75.4
G2 Blue Sky	24	−0.13	2.27	0.81	0.83	0.58	69.7
G3 Exoplan	23 *	−0.04	0.80	0.18	0.17	0.20	115.1

* Kruskal–Wallis test: *p* = 0.000 (significant differences). One Exoplan sample excluded due to sleeve fracture.

**Table 3:** Operative time (seconds) for apical trephination.

Group	Samples (n)	Minimum	Maximum	Median	Mean	SD	CV (%)
G1 Control	24	96.0	238.0	150.6	154.6	38.6	24.9
G2 Blue Sky	24	59.0	181.0	132.1	127.5	34.0	26.7
G3 Exoplan	23	54.0	135.0	114.8	106.5	22.8	21.4

Kruskal–Wallis test: *p* = 0.000 (significant differences).

## Data Availability

Data are available from the corresponding author upon reasonable request.

## Abbreviations

The following abbreviations are used in this manuscript:

CBCT	Cone-Beam Computed Tomography
GE	Guided Endodontics
SGE	Static Guided Endodontics
DGE	Dynamic Guided Endodontics
ICC	Intraclass Correlation Coefficient
SD	Standard Deviation
CI	Confidence Interval
ANOVA	Analysis of Variance
3D	Three-Dimensional
